# Relative performance of different exposure modeling approaches for sulfur dioxide concentrations in the air in rural western Canada

**DOI:** 10.1186/1471-2288-8-43

**Published:** 2008-07-04

**Authors:** Igor Burstyn, Nicola M Cherry, Yutaka Yasui, Hyang-Mi Kim

**Affiliations:** 1 Community and Occupational Medicine Program, Department of Medicine, Faculty of Medicine and Dentistry, The University of Alberta, Edmonton, Alberta, Canada; 2 Department of Public Health Sciences, School of Public Health, The University of Alberta, Edmonton, Alberta, Canada; 3 Department of Mathematics and Statistics, The University of Calgary, Calgary, Alberta, Canada

## Abstract

**Background:**

The main objective of this paper is to compare different methods for predicting the levels of SO_2 _air pollution in oil and gas producing area of rural western Canada. Month-long average air quality measurements were collected over a two-year period (2001–2002) at multiple locations, with some side-by-side measurements, and repeated time-series at selected locations.

**Methods:**

We explored how accurately location-specific mean concentrations of SO_2 _can be predicted for 2002 at 666 locations with multiple measurements. Means of repeated measurements on the 666 locations in 2002 were used as the alloyed gold standard (AGS). First, we considered two approaches: one that uses one measurement from each location of interest; and the other that uses context data on proximity of monitoring sites to putative sources of emission in 2002. Second, we imagined that all of the previous year's (2001's) data were also available to exposure assessors: 9,464 measurements and their context (month, proximity to sources). Exposure prediction approaches we explored with the 2001 data included regression modeling using either mixed or fixed effects models. Third, we used Bayesian methods to combine single measurements from locations in 2002 (not used to calculate AGS) with different *priors*.

**Results:**

The regression method that included both fixed and random effects for prediction (Best Linear Unbiased Predictor) had the best agreement with the AGS (Pearson correlation 0.77) and the smallest mean squared error (MSE: 0.03). The second best method in terms of correlation with AGS (0.74) and MSE (0.09) was the Bayesian method that uses normal mixture *prior *derived from predictions of the 2001 mixed effects applied in the 2002 context.

**Conclusion:**

It is likely that either collecting some measurements from the desired locations and time periods or predictions of a reasonable empirical mixed effects model perhaps is sufficient in most epidemiological applications. The method to be used in any specific investigation will depend on how much uncertainty can be tolerated in exposure assessment and how closely available data matches circumstances for which estimates/predictions are required.

## Background

It is well established that errors in exposure estimation can bias the results of epidemiological investigations. This takes most commonly the form of attenuation of the exposure-response association such that there is a danger of a false negative conclusion [1,2]. In addition, non-differential exposure misclassification can lead to reduced widths of confidence intervals of risk estimates, potentially leading to false positive results [1]. In some circumstances, differential misclassification of exposure can also produce positive bias in exposure-response relations, leading to false positive findings [3]. The implications of both false negative and false positive results of epidemiological studies can be profound. Specifically, in the first case, important causes of disease could be missed and, as a consequence, preventable disease may remain unchecked. In the second case, harm could be caused by implementation of inappropriate prevention measures and policies, and by creating unnecessary anxiety in the community.

In statistical literature, exposure misclassification and miss-measurement are known as a measurement error problem and a plethora of approaches exist to correct for biases that arise from it under certain assumptions [4,5]. One obvious approach to the problem is to obtain more precise exposure estimates instead of correcting for a known or suspected extent of exposure miss-measurement. In this regard, advances in monitoring technology have been helpful, such as passive monitoring that reduces the cost of measuring exposures, thereby obtaining larger volumes of relevant data that yield more accurate exposure estimates [6-9]. In the current project, passive monitoring technology was used to collect large quantities of air quality measurements over a vast geographical area.

In parallel, developments in exposure modeling/prediction methodologies are also valuable, such as group-based [10,11] and (statistical) model-based based exposure assessment [12], even though they are only recently starting to 'connect' with the mainstream literature on measurement error. Although the ecological fallacy may arise in epidemiological studies that utilize this approach, this does not diminish the utility of group-based exposure assessment in which all members of a group are assigned the same exposure status that reflects average exposure in the area/group. The ecological fallacy can be avoided by collecting information on confounders at the individual level. This approach to exposure misclassification is still under active development and there are ongoing arguments as to whether it is possible to infer individual exposure from either micro- (e.g. in persons' living room) or macro-environment (e.g. central air monitoring station for a town) measurements [13].

One of the exposure modeling approaches that, at least conceptually, holds great promise incorporates knowledge from empirical (statistical) and theoretical (physical) exposure assessment approaches in the Bayesian framework [14]. It has been suggested that, in occupational exposure assessment, a more accurate estimate of exposure can be obtained by combining pre-existing information about exposure status (e.g. schematics of workplaces, knowledge of chemicals used and transformed in a workplace, historical measurements from related operations, opinions of occupational hygienists) with exposure measurements [14]. This idea was critiqued [15] emphasizing that informative *priors *cannot be obtained in most occupational studies due to the lack of validated physical exposure models. However, the suggested approach may hold more promise in applications where informative *priors *can be obtained, as in modeling of air quality in relation to industrial emissions into the general environment or from routinely collected data on air quality, to provide some notion of the shapes of probability distributions of exposure in a given location.

Area measurement of air pollutants is often used as a proxy of exposure in epidemiological studies and for the purpose of this paper the two terms will be used interchangeably. The main objective of this work was to determine how we can best use currently available information on air concentrations of SO_2 _in rural western Canada to predict location-specific average exposure in a manner that is both cost-effective and accurate. We explore a prediction problem in a different time period at the fixed monitoring sites where some relevant data on sources and past air quality data may be available.

## Methods

### Data

**Figure 1:**
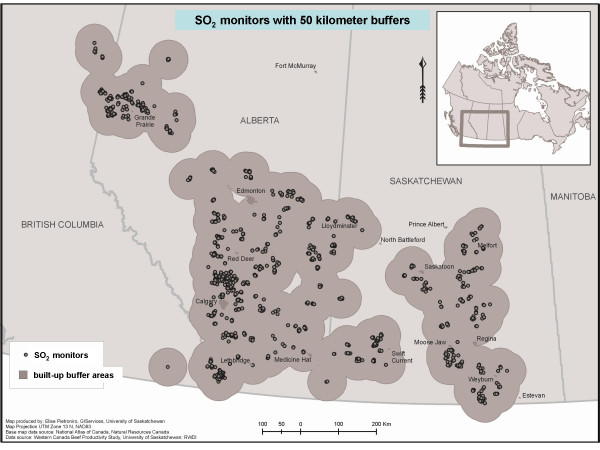
Maps of spatial distribution of SO_2 _air quality monitors.

Air monitoring data were collected for the study of health of cattle, as indicators of possible health effects on humans [16]. Month-long average air samples of SO_2 _(i.e. measurements integrating concentrations over a calendar month) were collected over a two-year period (April 2001 to December 2002) at various locations (Figure 1) across rural areas of western Canada that are associated with both cattle ranching and primary oil/gas exploration. In any given month, there were between 115 and 928 SO_2 _monitoring sites: the numbers of monitoring sites peaked in summer and declined in winter, primarily because monitoring sites tracked the movement of cattle herds, which were dispersed in summer and concentrated in winter. The proportion of sites with repeated measurements within a month (side-by-side measurements) was ~90% till August 2001, but then declined to ~10%. Air quality (SO_2_) measurements were described reasonably well by lognormal distributions. Air concentrations of SO_2 _in 2001–2002 (N = 13,991) had a geometric mean (GM) of 0.50 ppb and a geometric standard deviation (GSD) of 2.2. Air concentrations of SO_2 _in 2001 (N_1 _= 9,464) were somewhat lower on average (GM_1 _= 0.47 ppb) and less variable (GSD_1 _= 2.07) than in 2002 (N_2 _= 4,527, GM_2 _= 0.57 ppb, GSD_2 _= 2.37). The proportion of non-detectable measurements was low (a maximum of 2.5% in June 2002); these values were replaced by half of the detection limit (0.005 ppb) in all analyses.

**Figure 2:**
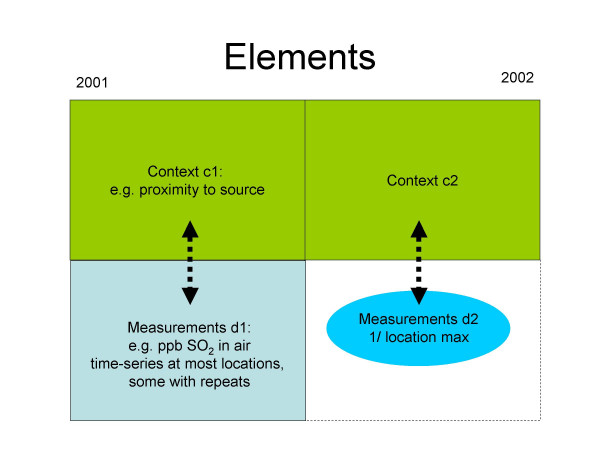
Data elements and their nomenclature.

For the purpose of this methodological investigation, we imagine that measurements were available only from 2001 and that our objective was to predict location-specific average exposures in 2002 (as was indeed the goal of the animal health study from which the data arose). Furthermore, we assume that for 2002 we had an option (though not necessarily exercised, depending in the hypothetical scenario outlined below) to collect one measurement from a randomly selected relevant (i.e. when cattle was housed at the site) month at each location. We will use nomenclature described in Figure 2 to refer to different data elements: d1 and d2 refer to measurements collected in 2001 and 2002, respectively; c1 and c2 refer to contextual data, such as month and proximity to oil and gas infrastructure, for each measurement in 2001 and 2002, respectively.

### Alloyed gold standard (AGS)

In order to evaluate the performance of different exposure modeling approaches, we need to know the true value of the location-specific mean exposure at each location in 2002. However, we only have observed time-series with repeated observations at each location and therefore can only estimate these values. Consequently, we were only able to assess the performance of different exposure modeling approaches in relation to our best estimate of the true value. This approach that does not adjust for measurement error and yet is free from any model assumptions is a location-specific *arithmetic *average, a direct measure of latent quantity of interest. We computed this at locations where there were repeated measurements in 2002 and designated it as M0*. Measurements that were imagined to have been collected in 2002 (d2) were the location-stratified random subset of all 2002 measurements; they were not used in calculation of the alloyed gold standard.

### Overview of prediction methods considered

One measurement from each location that was not used to calculate AGS was assumed to have been observed in 2002. We considered approaches that uses one month-long average measurement from each location of interest in 2002 (**M1**); and the other – context data on proximity of monitoring sites to putative sources of emission in 2002 (**M2**). In addition, we imagined that all of the previous year's (2001's) data were also available to exposure assessors. Exposure prediction approaches we explored with the 2001 measurement data included regression modeling using either mixed (**M3**) or fixed effects models (**M3f**). Lastly, we used Bayesian methods to combine single measurements from locations in 2002 (**M1**) with different *priors *(**M4-M6**). These approaches described within two separate scenarios below: without any measurements from 2002 (**M2**, **M3, M3f**) and with one measurement per location of interest in 2002 (**M1, M4-M6**).

### The first scenario: no measurements in 2002

If we choose not to collect any measurements in 2002 and rely on the 2001 data to make 2002 exposure predictions, we may consider two options. First, we could construct a model of the determinants of exposure using only 2001 data (d1 and c1). We will assume that it will have the same functional form as a model built previously [16]. We can then use *fixed *effect estimates of that model to estimate exposures in 2002 using context c2 for 2002 (method **M3f**) or use both *fixed *and *random *terms of the model to estimate exposures in 2002 using the 2002 distance to sources, context c2, to obtain Best Linear Unbiased Predictors, (BLUP) (**M3**).

The following model of the determinants of exposure could be constructed using the 2001 data (d1 and c1 only):

(1) ln(SO_2_, ppb) = -0.97+0.26ln [Σ_allΔ2_(Δ_2 _oil wells)^-2/3^]+0.24ln [Σ_allΔ2-50_(Δ_2-50 _oil wells)^-2/3^] +12.33ln [Σ_allΔ2_(Δ_2 _gas plants)^-2/3^]+4.15ln [Σ_allΔ2-50_(Δ_2-50 _gas plants)^-2/3^]+random effects,

where Δ_2 _= distance in km from the monitoring location to a specified oil and gas infrastructure (oil wells or gas plants in this case) within the 2 km radius of the monitoring station (industrial infrastructure outside of this radius was ignored in the calculation of Δ_2_); Δ_2-50 _= distance in km from the monitoring location to a specified oil and gas infrastructure within the 2–50 km torus; and random effects with the estimate of between-location variance (s^2^_L1_) 0.23, the estimate of month-to-month variance (s^2^_T1_) 0.09, and the estimate of between-repeat (within month and location) variance (s^2^_R1_) 0.21. This model is very similar in terms of the magnitude of fixed and random effects to the model that was previously derived in the basis of the entire data available to us [16]. The rationale for formulating distance to sources as in equation (1) is described in greater detail below.

Alternatively, we could be skeptical about the value of 2001 data and models that they yield, and rely exclusively on the description of measurement sites in 2002 in terms of their proximity to oil and gas infrastructure (i.e. c2) to rank locations in terms of expected SO_2 _concentrations (**M2**). Several such rankings are possible, because we do not know *a priori *which context (i.e. proximity to what type(s) of facilities) is best to use. Concentrations near point sources of emission in flat terrain without strong prevailing winds can be described as being directly proportional to the emission rate and inversely proportional to the separation distance taken to the power of 2/3, a distance decay model [17]. This informed the parameterization of predictive models we developed in **M3**, and appears to be a reasonable starting point for ranking different monitoring sites with respect to anticipated air quality. However, there is uncertainty about which distance to which oil and gas facilities is the most sensible to use in predicting SO_2 _concentrations. On one hand, strong sources of SO_2 _emissions, such as gas plants, seem obvious candidates, but they are less numerous and farther away from monitoring locations than wells and batteries. Thus, all these facilities can potentially impact SO_2 _concentration and the context of 2002 measurement sites (c2) was described in terms of proximity to all wells, all batteries and all gas plants. The proximity measure was described in detail previously [16]: it is a sum of (distance in km)^-2/3 ^for each facility type within 2 km or 50 km radius around each monitoring site. The coordinates of different active oil and gas facilities in 2002 were supplied to us by the regulatory agencies from the Canadian provinces of Alberta, Saskatchewan and British Columbia, enabling us to estimate the distances. Proximity to the following facilities was estimated: wells with 2 km, wells within 50 km, batteries within 2 km, batteries within 50 km, gas plants within 2 km, and gas plants within 50 km.

All regression models and their predictions in the manuscript were made in SAS (version 9.1, SAS Institute, Cary, NC) PROC MIXED using the REML algorithm.

### The second scenario: some measurements in 2002

If one measurement was collected in 2002 from each location of interested on a randomly chosen month (d2), we can consider the following exposure estimation options. A simple approach is to use a single measurement from each location in 2002 to estimate mean location-specific exposures in 2002 (**M1**).

We could also dismiss the 2001 data except for estimating measurement error variance using repeated measurements and then 'correct' 2002 measurements for this measurement error under the assumption of log-normal distribution of true exposure levels (**M4**). We can also use estimates from **M3 **as *a basis for an empirical normal mixture prior *with an *unknown number of components *for observed data d_2 _to obtain method **M5**. Alternatively, we could mistrust 2001 measurements and rely only on the context of 2002 measurements (c2) for the prior information, leading us to method **M6**, which also utilizes *normal mixture prior *with an *unknown number of components*.

Bayesian approaches have been adopted for adjusting bias arising from measurement error [5]. Parameters of a Bayesian model are not assumed to be fixed, but vary at random in accordance with some probability distributions. For each parameter (or a set of parameters), a probability distribution that reflects its prior knowledge/belief is specified and combined with the likelihood function of the data to obtain a posterior distribution of the parameter(s) (e.g., location-specific means of SO_2 _concentration in our case). This posterior distribution includes all knowledge/belief related to the parameters from the prior and the likelihood involving covariates (i.e. data and assumed models). It is usually obtained by means of the Monte Carlo integration using Markov Chain (MCMC) unless it is analytically tractable. The variables observed with error are also considered to be random, so that they are incorporated into the process of sampling from the posterior.

Bayesian analysis has been developed to adjust for measurement error by specifying two sub-models: i) a measurement error model relating the observed exposure with error and the true exposure; and ii) the *prior *distribution of the true exposure. The true exposure is assumed to have either a lognormal distribution for a specific known *prior *(**M4**), or a mixture of normal distributions with unknown number of components, a flexible approach aimed to overcome potential misspecification of the *prior *distribution (**M5 **and **M6**). The reversible jump algorithm [18] is used for the normal mixture prior with unknown number of components, together with the standard Gibbs or Metropolis algorithm. The details of the Bayesian models and their implementation are given in the Appendix.

In implementing **M4 **(in *R*: Copyright 2005, The R Foundation for Statistical Computing Version 2.1.1 (2005-06-20), ISBN 3-900051-07-0), we obtained an MCMC chain with 45,000 iterations and discarded the first 15,000 'burn-in' interactions. In implementing **M5 **and **M6 **(in FORTRAN), we used 100,000 'burn-in' iterations and used the subsequent 100,000 iterations to obtain estimates of posterior for each location.

### Measures of relative performance

Comparing estimated exposures to M0* (the arithmetic mean used as the AGS) will enable us to evaluate relative performance of different exposure assessment methods. In environmental epidemiology, the association of interest may be that between the concentrations of a contaminant (ppb SO_2 _in our case) and risk of a disease. The most commonly-used exposure-disease model is the logistic regression model. Because the relationship between true (ϕ_T_) and observed (ϕ_O_) risk gradients in logistic regression is determined by Pearson correlation between true and observed exposure (ρ_TO_) as in ϕ_T _= ϕ_O_/ρ^2^_TO _[1], and a correlation between two random variables can be estimated without fully specifying their distributions, we use the Pearson correlation between the SO_2 _levels predicted by the different exposure estimation procedures and the alloyed gold standard (M0*) as a measure of relative performance of the different procedures. We also computed mean squared error (MSE): mean of (estimate – AGS)^2^.

## Results

The alloyed gold standard could only be calculated for the 666 sites that had repeated air quality measurements (out of total of 903 sites) in 2002. The average number of repeated measurements per location was six, ranging from two to 24.

**Figure 3:**
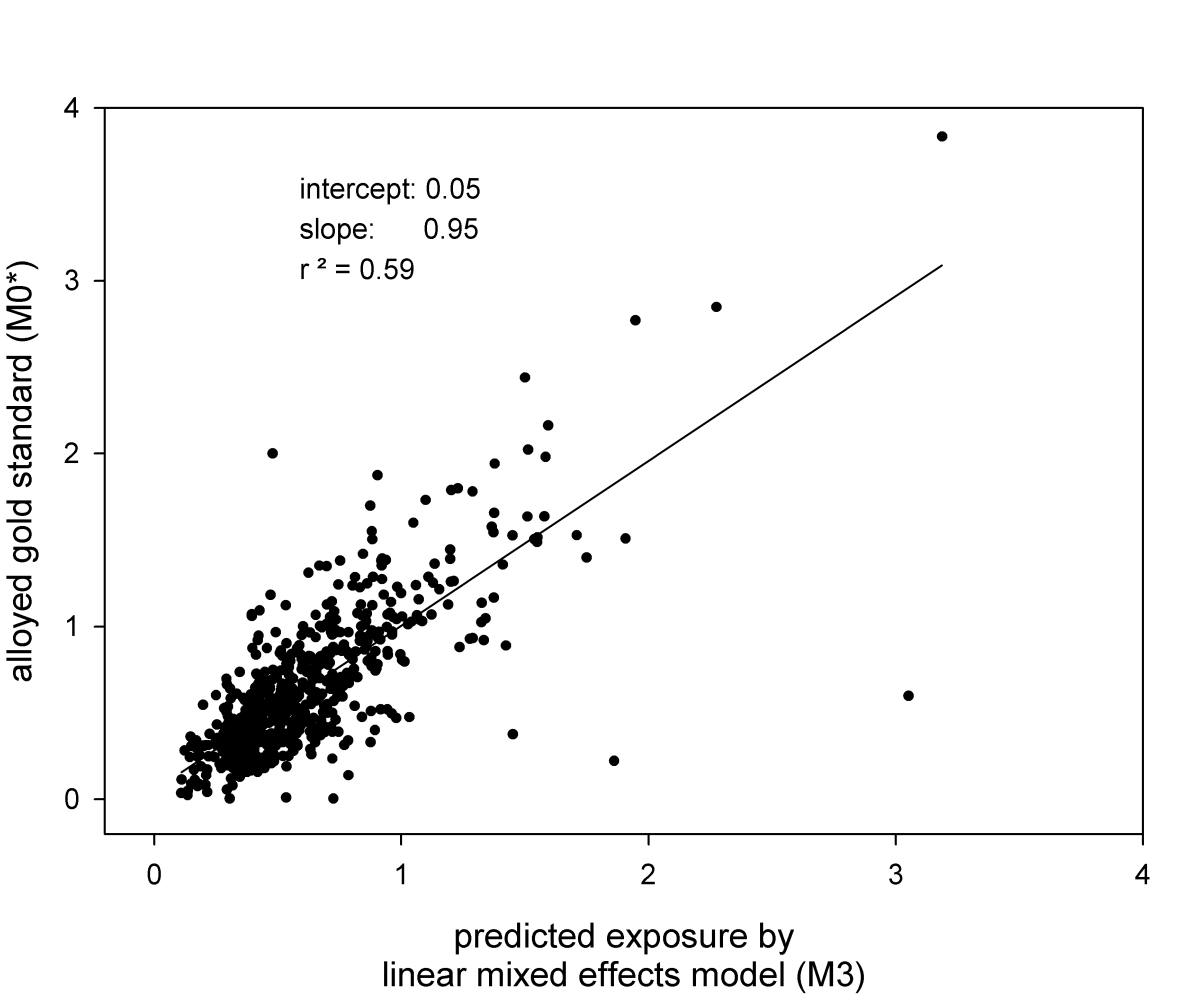
Agreement between the alloyed gold standard (M0*) and predictions on the basis of linear mixed effects model (BLUP, M3), N = 666, each axis is in the units of ppb of SO_2_.

**Figure 4:**
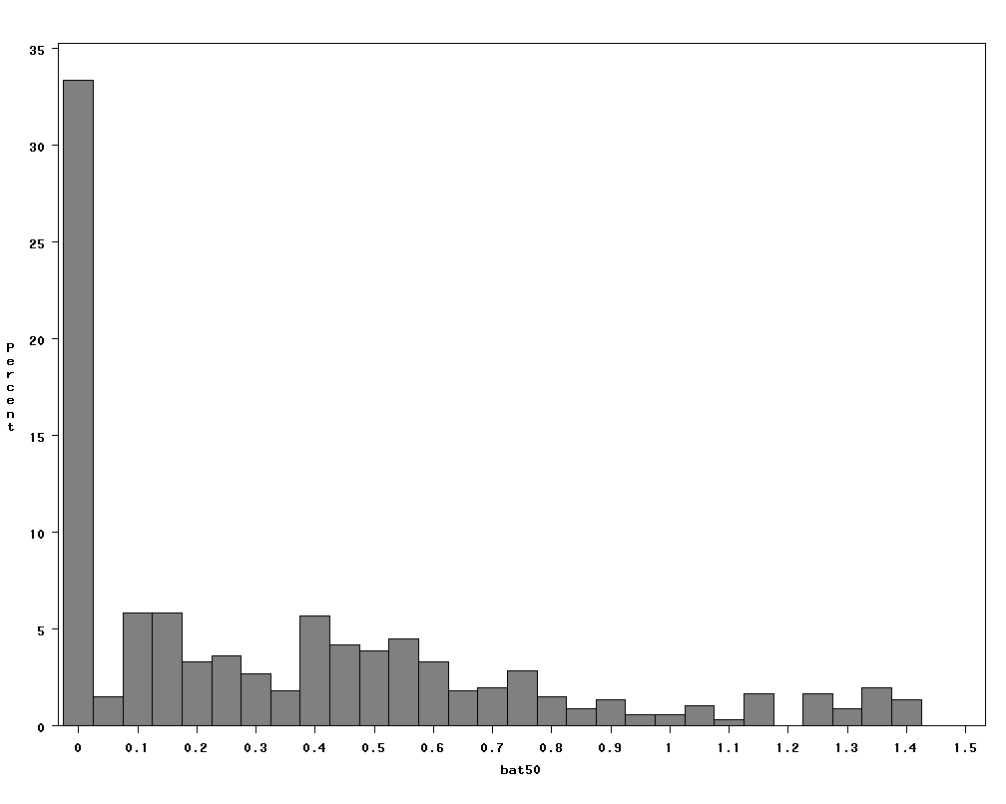
Histogram of measures of proximity to all (oil and gas) batteries within 50 km radii (bat50): prior for method 6 (N = 666); x-axis: proximity to batteries with 50 km of the monitor (km^2/3^).

A summary of the relative performance of the different exposure estimation methods is presented in Table 1. Overall, **M3 **appears to be superior in terms of the strongest correlation with the alloyed gold standards and the smallest MSE (Figure 3). Recall that in **M3**, we used both fixed and random terms of the model based on 2001 data to predict 2002 measurements (by plugging-in functions of distance to sources, c2, into equation (1) and computing mean SO_2 _concentration (ppb) for each location). If only the fixed effects from equation (1) were used (as is the case if a fixed-effects ordinary least square model was used to identify determinants of exposure), a poor agreement between predicted (**M3f**) and the alloyed gold standard was observed (r = 0.33). The application of a distance-decay model [17] in the exposure estimation method **M2 **produced the worst predictions: only the correlation with proximity to all batteries within 50 km was positive and statistically different from zero: r = 0.21 (MSE = 0.28). Proximity to batteries was selected as a *prior *for **M6**, because its correlation is the only consistent positive predictor of measured SO_2 _levels (**M2**), and because earlier work relied on the assumption that batteries are a good proxy of exposure to SO_2 _[19], making it a natural choice for the Bayesian *prior *derived from c2. The distribution of values used as a *prior *based on proximity to batteries (also equivalent to **M2**) is shown in Figure 4; it implies a distribution that does not easily fit any common parametric form. The use of this *prior *with measurements collected in 2002 in the Bayesian normal mixture method (**M6**) produced estimated SO_2 _concentrations that did not agree very well with the alloyed gold standard: r = 0.28 (MSE = 0.30). The normal mixture approach with a *prior *derived from air quality predictions obtained in **M3 **(**M5**) yielded the second best predictions.

**Table 1:** Comparison to alloyed gold standard constructed as a mean of observed measurements from a given location in 2002 when there were at least two measurements (2 to 24; average = 6, N = 666).

**Exposure Assessment Method for annual mean in 2002**			**ρ_TO_^a^**	**MSE^b^**
	
Type of method/model	Model description^**Nomenclature**^	Use of measurements^c^		
*No model*	one measurement per location^**M1**^	2002	0.67	0.15
*Distance-decay*	contextual data only^**M2**^	None	0.21	0.28
*Regression*	*Effects used in prediction*			
	fixed & random, BLUP^**M3**^	2001	0.77	0.03
	fixed effects^**M3f**^	2001	0.33	0.12

*Bayesian*	*Prior*			
	lognormal^**M4**^	2001 & 2002	0.68	0.15
	normal mixture from regression model M3^**M5**^	2001 & 2002	0.74	0.09
	normal mixture from context, M2^**M6**^	2002	0.28	0.30

a: correlation with alloyed gold standard; b: mean squared error; c: only one measurement per location in 2002

## Discussion

Strictly speaking, our observations only apply to the particular data set from which they were derived and a specific sample of 2002 observations. However, the results suggest some general conclusions about the estimation of environmental concentrations of pollutants derived from industrial sources. When no measurements of air quality are available, we can expect predictions by a simple distance-decay model to have poor agreement with true air quality (**M2**). When only a relatively small measurement effort is possible in the time-period of interest and the magnitude of measurement error is known from some validation studies, the empirical Bayesian methodology that relies only on 2002 data (d2) and some estimate of measurement error (**M4**) produced results that were not markedly different from just using one measurement per location to estimate true location-specific concentrations (**M1**). However, if a very poor prior (**M2**) is combined with a limited set of exposure measurements (**M1**), even if these measurements are close to 'true' values, the Bayesian methodology leads to inferior estimates of true values (**M6**). The poor *prior *appears to degrade advantages present in the data.

When only exposure measurements collected from adjacent time and places of interest are available, we can expect to obtain reasonable estimates if we rely on the empirical BLUP of the mixed effects models (**M3**), not just predictions based on estimates of fixed effects (**M3f**). The Bayesian normal mixture method with flexible *prior *also seems to have a reasonable performance (**M5**), especially if one considers pitfalls inherent in the alternative approaches. Namely, **M3 **will perform poorly if there is a large change in air quality between 2001 and 2002, but **M5 **would utilize 2002 data preferentially and be less affected by this. However, as suggested by results with 'poor' *prior *(**M6**), when there is a large difference in exposure between the data sets used to model exposure and true exposure being predicted, the Bayesian normal mixture method is expected to falter relative to the simple collection of relevant data. This echoes a previous suggestion that, in many situations, the effort involved in modeling exposures may exceed that required to collect measurements [20].

Methods **M1 **and **M4 **had virtually identical agreements with the alloyed gold standard, which was inferior to methods **M3 **and **M5**. We can ascribe poor performance of **M1 **to failure to account for measurement error, since it uses only one observation per location in 2002, and ignoring the context of 2002 exposures. In Bayesian method with lognormal *prior *that uses 2001 data only to define measurement error variance (**M4**), inferior predictions can be ascribed to improper *prior *specification, an extreme case of poor *prior *also illustrated by **M6**, as well as ignoring the context of 2002 exposures. This suggests that methods that fail to correct for measurement error and/or are based on poor *priors *can be expected to yield predictions of inferior accuracy.

The main limitation of our study is the lack of a gold standard to evaluate the performance of different exposure assessment procedures. We are inclined to believe that our choice of gold standard that is free from model assumptions is indicative of true performance of the compared methodologies. In this way, comparison is not biased in favor of a method that may be employed to produce an alloyed gold standard adjusted for measurement error. Thus, although our chosen alloyed gold standard is contaminated by measurement error, it was obtained without resorting to the assumptions that are used in the competing exposure assessment methods.

We had the luxury of a large 2001 dataset that enabled us to create an empirical *prior *that probably closely reflects the distribution of true values and the extent of measurement error. It may not be possible to rely on such pre-existing data in many studies. Given the sensitivity of the Bayesian methods to 'quality' of the *prior*, careful judgment is required in deciding whether it is better to invest resources into extensive data collection or complex modeling. It must be noted that our 2001 data did not cover every month (data collection began in April) whereas 2002 measurements were spread across all months in 2002. This presented a realistic challenge to our exposure assessment models of estimating exposures for temporally misaligned data in presence of temporal trends in exposures within a year (see Figure 4 in [16]).

Our data was not very variable and contained only a modest measurement error. Thus, our conclusions may not hold for more variable and more error-prone situations that may arise in environmental exposure assessment, as reported for volatile organic compounds [21,22].

Another limitation of our work presented here is that we were not able to explore all possible modeling techniques that may be potentially available for predicting air pollution levels. It is for this reason that we focused on methods that appear to be sensible "first choices" in the given setting plus some more exotic Bayesian model that we wished to evaluate. Specifically, an autoregressive integrated moving average (ARIMA) approach may be suitable for part of our data where spatially aligned time-series can be identified as may be a more flexible methodology of Calder et al[23,24]. In addition, it may be possible to obtain better predictions through the empirical regression models by relaxing assumptions based on the model of Strosher[17], by either modeling the power transformation, employing generalized additive models, or using neural networks that relax parametric assumptions about the shape of distance-concentration association (see the Schlink et al[25] for overview of various other modeling options). We are exploring the utility of some of these modeling approaches in the current dataset in our parallel ongoing research.

## Conclusion

Initial large measurement efforts are unavoidable when characterizing air quality and evaluating various exposure assessment options. However, once a considerable amount of information has been obtained about a defined area and a particular contaminant, subsequent air quality surveys can be less costly and extensive if they utilize either regression BLUP (**M3**) or generate an empirical *prior *in regression BLUP to be followed by Bayesian exposure assessment that integrates prior knowledge with a limited series of new measurements (**M5**). On theoretical grounds, we prefer Bayesian approach **M5 **because it forces investigators to make weaker assumption about the distribution of true exposure and shows good performance in our situation. However, it places extra demands on both data collection and modeling efforts and, despite its theoretical advantage, failed to outperform the more straightforward BLUP method in our study. Whether the *priors *based on dispersion or distance-decay models prove to be useful remains to be determined, but our findings are not encouraging. It is likely that either collecting some measurements from the desired locations and time periods (**M1**) or predictions of a reasonable empirical mixed effects model perhaps (**M3**) is sufficient in most applications. Furthermore, the simplicity of **M3 **relative to **M5**, without obvious gains in accuracy, would probably make **M3 **the pragmatic choice in many settings. The method to be used in any specific investigation will depend on how much uncertainty can be tolerated in exposure assessment and how closely available data matches circumstances for which estimates/predictions are required.

## Competing interests

The authors declare that they have no competing interests.

## Authors' contributions

IB and NMC developed research proposal, which was refined in consultations with YY and H–MK. H–MK developed and implemented algorithms required implementing Bayesian methods. All authors contributed equally to writing the manuscript; they read and approved the final manuscript.

## Appendix: Details of Bayesian methods M4, M5 and M6

True exposure *X *is observed with error as *U*. The goal of the methods presented below is to estimate *X *on the basis of *U *using information and assumptions about the nature of the measurement error.

In applying the method **M4**, we specify the two sub-models:

*p*(*U*_*i *_| *X*_*i*_, *λ*) : measurement error model

*p*(*X*_*i *_|*π*): prior (true exposure) model for *X*_*i*_

and the joint distribution of *X*_*i *_and *U*_*i *_is $p\left(\lambda \right)p\left(\pi \right)\prod_{i}p\left(X_{i}|\pi \right)\prod_{i}p\left(U_{i}|X_{i},\lambda \right)$, where *p*(*λ*) and *p*(*π*) are the prior distributions for the parameters of the two sub-models, and p(• | •) to denote generic conditional distributions consistent with the joint specification.

The measurement error model for *U*_*i *_conditional on *X*_*i *_is given by log (*U*_*i*_) ~*N*(log(*X*_*i*_), *τ*^2^), where *λ *= *τ*^2 ^is known and the prior for a lognormal distribution is given by *X*_*i*_~log *N*(*μ*, *σ*^2^), where *π *= (*μ*, *σ*^2^). The parameters *μ *and *σ*^2 ^are assumed to have a normal distribution with mean 0 and a variance *s*^2 ^(sample variance) and a highly dispersed inverse gamma distribution with parameters 1 and 0.005, respectively. We derive full conditionals for the parameters as follows:

*X*_*i *_| *rest *~*N*(*μ*, *σ*^2^)

$$\mu |rest\sim N(\frac{s^{2}\sum \log\left(x_{i}\right)}{s^{2}n+\sigma^{2}},\frac{s^{2}\sigma^{2}}{s^{2}n+\sigma^{2}})$$

$$\sigma^{2}|rest\sim IG(\frac{n}{2}+1,\frac{1}{2}{\left(\log\left(x_{i}\right)-\mu \right)}^{2}+0.005)$$

We use a Metropolis-Hastings algorithm with a random walk proposal to first update *X*_*i *_and then *μ *and *σ*^2 ^in each step. Initial values of *σ*^2 ^come from the logarithmic variance of the distribution of 2002 measurements (d2) and *τ*^2 ^is the variance between repeats of 2001 data, s^2^_R1 _(see above).

In applying the methods **M5 **and **M6**, we follow Richardson and Green [26], and use a mixture of normal distributions with unknown number of components as a prior model for *p*(*X*_*i *_| *π*):

$$X_{i}\sim \sum_{j=1}^{k}\omega_{j}f\left(\cdot |\theta_{j}\right)\text{ independently for }i=1,\cdots ,n$$

where *f *(· | *θ*) is a normal distribution. The unknown number of k components with parameters *θ*_*j *_= (*μ*_*j*_, *σ*^2^_*j*_) and the components weights *ω*_*j *_summing up to 1 are unknown.

The hierarchical formulation of this mixture model introduces latent allocation variable *z*_*i *_that indicates to which mixture component the observation *X*_*i *_belongs. This model can be formulated by:

*p*(*z*_*i *_= *j*) = *ω*_*j *_independently for *j *= 1, 2, …, *k *and given the value of the *z*_*i*_, $X_{i}|z\sim f\left(\cdot |\theta_{z_{i}}\right)$ independently for *i *= 1,2, …, *n*.

We use the same notation for the conditional distributions, and $\omega ={\left(\omega_{j}\right)}_{j=1}^{k}$, $z={\left(z_{i}\right)}_{i=1}^{n}$, $\theta ={\left(\theta_{j}\right)}_{j=1}^{k}$, $X={\left(X_{i}\right)}_{i=1}^{n}$ and $U={\left(U_{i}\right)}_{i=1}^{n}$. The joint distribution is given by

*p*(*k*, *ω*, *z*, *θ*, *τ*^2^, *X*, *U*) = *p*(*k*)*p*(*ω *| *k*)*p*(*θ *|*z*, *ω*, *k*)*p*(*z *| *ω*, *k*)*p*(*X *|*θ*, *z*, *ω*, *k*)*p*(*U *| *X*, *τ*^2^), which is equivalent to *p*(*k*, *ω*, *z*, *θ*, *τ*^2^, *X*, *U*) = *p*(*k*)*p*(*ω *| *k*)*p*(*z *| *ω*, *k*)*p*(*θ *| *k*)*p*(*X *|*θ*, *z*)*p*(*U *| *X*, *τ*^2^) by imposing independence assumptions, *p*(*θ *|*z*, *ω*, *k*) = *p*(*θ *| *k*) and *p*(*θ *| *z*, *ω*, *k*) = *p*(*X *|*θ*, *z*).

We allow the priors for *k*, *ω *and *θ *to depend on hyper-parameter *λ*, *δ*, *η *= (*ξ*, *κ*, *α*, *β*), respectively, and specify *priors *as *μ*_*j *_~*N*(*ξ*, *κ*^-1^), $\sigma_{j}^{2}$ ~Γ(*α*, *β*), *k *~*Poisson*(*λ*), *ω*~*Dirichlet *(*δ*_1_, *δ*_2_, *δ*_*k*_), and *β *is the only hyper-parameter which is not treated as fixed, being given a gamma distribution with parameter *g *and *h*. The full conditional distributions for parameters are following.

$$X_{i}|rest\sim N(\mu_{z_{i}},\sigma_{z_{i}}^{2})$$

*ω *| *rest *~*D*(*δ *+ *n*_1_, …, *δ *+ *n*_*k*_)

$$\mu_{j}|rest\sim N[\frac{\sigma_{j}^{-2}\sum x_{i}+\kappa \xi }{\sigma_{j}^{-2}n_{j}+\kappa },\frac{1}{\sigma_{j}^{-2}n_{j}+\kappa }]$$

$$\sigma_{j}^{-2}|rest\sim \Gamma [\alpha +0.5n_{j},\beta +0.5\sum {\left(x_{i}-\mu_{j}\right)}^{2}]$$

$$p(z_{i}=j|rest)\propto \frac{\omega_{j}^{2}}{\sigma_{j}^{2}}\exp\left(-{\frac{\left(x_{i}-\mu_{j}\right)}{2\sigma_{j}^{2}}}^{2}\right)$$

$$\beta |rest\sim \Gamma (g+\kappa \alpha ,h+\sum \sigma_{j}^{-2})$$

We make use of 'moves' to update parameters:

1. updating *X *using (*z*, *θ*_*z*_, *U*) for corresponding to the individuals

2. updating the weight (*ω*, *z*, *θ*) conditional on *k*

3. updating the parameter *k *and consequently the relevant mixture parameters

The moves for updating the mixture parameters and changing *k*, the number of components by using reversible jump split/merge proposals, have been described in detail in Richardson and Green [26].

## Pre-publication history

The pre-publication history for this paper can be accessed here:


